# Suicidal behaviors in mental illness: A case-control study. Suicidal behaviors in mental illness: A case-control study

**DOI:** 10.1192/j.eurpsy.2024.1001

**Published:** 2024-08-27

**Authors:** A. Boumnijel, S. Rouached, S. Boudriga, M. Lagha, W. Homri, I. Ben Rhomdhane, R. Labbane

**Affiliations:** Department of Psychiatry C, Razi Hospital, Mannouba, Tunisia

## Abstract

**Introduction:**

The assessment of suicide risk remains a critical concern, especially within the psychiatric community. Mental health professionals continually work to identify and support individuals at risk, emphasizing the need for ongoing research and training in this area.

**Objectives:**

The objectives of our study were to understand the characteristics of patients hospitalized after a suicide attempt (SA), analyze the characteristics of these attempts, identify risk factors associated with suicidal behaviors, and determine predictors for recurring suicidal behavior.

**Methods:**

The study’s methodology was retrospective, descriptive, and comparative. It was conducted with 277 patients hospitalized in the psychiatric department “C” of Razi Psychiatric Hospital in Manouba. The sample consists of 72 individuals who attempted suicide, divided into two groups: first-time attempters and recurrent patients, and 205 controls hospitalized for other reasons during the same period.

**Results:**

Results showed a significant increase in the frequency of hospitalizations for SA, rising from 0.7% to 2.25% of the total admissions between 2018 and 2022. Those who attempted suicide were on average 32.5 years old, predominantly female, urban residents, with a moderate socioeconomic status, secondary or higher education, unemployed, unmarried, childless, and lacking strong family support.

The study identified several risk factors associated with suicide attempts, including risky behaviors, previous life events, type II bipolar disorders, personality disorders, the number of psychiatric hospitalizations, and the quality of follow-up. However, schizophrenia was negatively correlated with SA.

Suicidal recurrence was observed in 65.5% of attempters and was linked to personal psychiatric follow-up history, mood disorders, personality disorders, the presence of stress factors, and caustic substance ingestion.

**Conclusions:**

In conclusion, the study underscores the importance of assessing suicide risk among individuals with mental disorders to implement appropriate prevention strategies.

**Disclosure of Interest:**

None Declared

